# Repeated Injections of Iodine in the Treatment of Encysted Tumors

**Published:** 1851-03

**Authors:** 


*Repeated Injections of Iodine in the Treatment of Encysted Tumors*—M. Borrelli of Turin, makes a small oblique opening into the cyst, by which he evacuates its contents. He then injects, by means of a syringe, an alcoholic solution of iodine, which he leaves in the cyst, closing the aperture with diachylon or charpie. Pain ensues in the course of two or three minutes, and continues for about twenty-four hours. The inflammation which is excited may usually be allayed by a poultice. The tumor which before was tense and painful becomes softened and diminished in size, while a colored fluid oozes from the incision. Sometimes, after the first injection, and as soon as the local inflammation has subsided, the cyst becomes detached, and may be extracted with the forceps; but usually it is necessary to repeat the injections two or three times before obtaining a complete separation. When the cyst is withdrawn, the radical cure is easily completed.—*Lond. Med. Gaz. Dec.* 20*th*, 1850.

